# Preparation and Characterization of Eco-Friendly Mg(OH)_2_/Lignin Hybrid Material and Its Use as a Functional Filler for Poly(Vinyl Chloride)

**DOI:** 10.3390/polym9070258

**Published:** 2017-06-30

**Authors:** Łukasz Klapiszewski, Jolanta Tomaszewska, Katarzyna Skórczewska, Teofil Jesionowski

**Affiliations:** 1 Institute of Chemical Technology and Engineering, Faculty of Chemical Technology, Poznan University of Technology, Berdychowo 4, PL-60965 Poznan, Poland; teofil.jesionowski@put.poznan.pl; 2 Faculty of Chemical Technology and Engineering, UTP University of Science and Technology in Bydgoszcz, Seminaryjna 3, PL-85326 Bydgoszcz, Poland; katarzyna.skorczewska@utp.edu.pl

**Keywords:** lignin, magnesium hydroxide, magnesium hydroxide/lignin hybrid filler, poly(vinyl chloride), composite characterization

## Abstract

A functional magnesium hydroxide/lignin hybrid system was prepared by grinding and mixing the pure precursors using a planetary ball mill. In addition, the thermal stability was assessed based on the total mass loss of the hybrid system within the temperature range of 30–1000 °C, which amounted to 38%. Moreover, the average particle size was at 4.9 μm as determined by the laser diffraction method. The effect of addition of the prepared and characterized Mg(OH)_2_/lignin hybrid filler at concentrations ranging from 2.5 wt % to 10 wt % on the processing as well as mechanical and thermal properties of composites on the matrix of the unplasticized PVC compound was also evaluated. The addition of a filler to the poly(vinyl chloride) matrix causes a significant improvement of its thermal stability, which is approximately three times higher compared to a polymer without a filler. Furthermore, the prepared composites are additionally characterized by advantageous mechanical properties, especially higher Young’s modulus. A 10% increase in the oxygen index of PVC composites upon addition of 10 wt % hybrid fillers has also been observed, which contributes to an extended range of their application under conditions that require notable fire resistance.

## 1. Introduction

Composite materials with the addition of functional fillers, including hybrid materials, are used in almost all areas of everyday life. They have well-defined, enhanced mechanical, thermal and physicochemical properties; low specific gravity; and considerable chemical resistance [1,2,3,4,5,6].

Magnesium hydroxide belongs to a group of unique inorganic compounds, which exhibit numerous advantageous properties and practical applications. It is distinguished primarily by its antibacterial activity, nontoxic nature and high thermal stability. All of the above features make Mg(OH)_2_ an agent commonly used to reduce the combustibility of polymer materials [7,8,9,10,11,12,13,14].

Qiu et al. [7] proposed the use of magnesium hydroxide in the form of Mg(OH)_2_/vinyl acetate nanocomposite as a material with advantageous mechanical, reinforcing and non-combustible properties. Based on the obtained TEM images, a very high dispersion degree of the inorganic filler in the inorganic modifier was observed. The improvement of non-combustible properties of the formed composite was confirmed by comparing the determined Limiting Oxygen Index (LOI) values for both micro-Mg(OH)_2_/EVA as well as nano-Mg(OH)_2_/EVA. Based on the results, an increase in the oxygen number from 24.0 to 38.3 was noted for the material with nanometric refinement. The improvement of the non-combustible properties occurred due to the good dispersion of Mg(OH)_2_ in the EVA matrix and the formation of compact, charred structures during the combustion of the material, which showed similarity to the structure of ceramic materials.

In other studies, Chen et al. [8] carried out an experiment with the addition of Mg(OH)_2_ modified using zinc titanate and stearate, as a potential HFFR (Halogen Free Flame Retardant)-type filler applied to polypropylene (PP). The observations of the fillers microstructure using SEM images showed a better distribution of Mg(OH)_2_ particles after introduction of the titanate and stearate, in contrast to Mg(OH)_2_ with no modifier. The inclusion of the modifying compounds also favorably influenced the adhesion of the PP and Mg(OH)_2_ components, directly contributing to the improvement of their compatibility, and thus the mechanical properties of the formed PP/Mg(OH)_2_ composite. The obtained tensile strength value for PP/Mg(OH)_2_/zinc titanate and PP/Mg(OH)_2_/zinc stearate was higher by approximately 6–8 MPa compared to PP/Mg(OH)_2_ (i.e., by 35–45%). In turn, the results of composite flame retardancy tests showed that an increase in the oxygen index value occurred as a result of increased modifier content.

Using Mg(OH)_2_ as a fire retardant was also reported in study [9], which confirmed the existence of a synergistic effect between sepiolite (a mineral from the group of silicates) and magnesium hydroxide in the formed composite, with a LLDPE matrix. Suihkonen et al. carried out investigations to determine the flame retarding effect of magnesium hydroxide on epoxy (EP) resins [10]. The study determined the effect of the grain size of Mg(OH)_2_ and the modification of its surface with silanes on the thermal and mechanical properties of EP components. Both the nano- and micro-particles of Mg(OH)_2_ used in the experiment were modified using 3-aminopropyltriethoxysilane in a water solution, and were used as fillers introduced to the epoxy matrix. The obtained test results confirmed that nano-Mg(OH)_2_ introduced improved mechanical properties to EP, compared to its counterpart on the micrometric scale. In his study, Wang demonstrated that modified Mg(OH)_2_/siloxane nanocomposites obtained using the hydrothermal and hypergravity method also had potential flame retarding properties [11]. The results showed that the stability of the prepared magnesium hydroxide/siloxane nanocomposite flame retardants was superior to Mg(OH)_2_ particles obtained by other methods, and the agglomeration was significantly reduced. The surface area of the composite particles was reduced, and the affinity and thermal stability were effectively improved. As a type of filler for polymers, the Mg(OH)_2_/siloxane nanocomposite may improve the reinforcement of the polymer and the heat resistance and flame retardancy, which provide the foundation for further application of this nanocomposite as a flame retardant. Ma et al. used magnesium hydroxide by applying it onto the surface of cotton fibers with the aim of reducing the combustibility and improving the thermal stability of cotton/magnesium hydroxide composite [12]. Excellent mechanical and thermal properties were achieved in this system, which are rarely obtained in case of polymers. In case of the reported tests, advantageous surface changes occurred (i.e., the formation of a larger quantity of Mg(OH)_2_ crystals) as a result of modification with urea and a citric acid solution. On the other hand, the composite combustibility tests showed that introduction of sodium chloride to the cotton/magnesium hydroxide composite considerably increased the fire resistance of the fabric. These results may also be applied for development of new polymer/inorganic material composites.

Within the framework of the conducted research, it seems reasonable to also use poly(vinyl chloride) (PVC), which is one of the most important engineering polymers. Its production is constantly increasing due to numerous applications. Among other thermoplastic polymer materials, PVC is distinguished by the possibility to modify its properties over a wide range by using many additives, including fillers [13,14]. This applies to both processing and functional properties, the latter of which enable very diverse applications of this polymer. In view of the very long life of PVC products, the greatest share of the consumption of elements made of unplasticized PVC falls to the building industry. The versatility of the application of PVC is mainly associated with its very good resistance to environmental factors and advantageous mechanical properties [15,16]. Commonly used rigid PVC products include, above all, window framing and furniture sections, external façade elements, water supply and sewage tubing, plates, sheets, etc.

One of the reasons for the widespread use of PVC in the building industry is the fact that this polymer is classified as the only non-combustible material among general-purpose polymeric materials, due to its chloride content (above 56%). Furthermore, PVC is characterized by the lowest value of heat of combustion [17,18] compared to other polymer materials, which notably limits the risk of fire.

Although many common applications of rigid PVC do not require the use of flame retardants, investigations are undertaken to additionally enhance its fire resistance and reduce its smoke generation ability. This results from increasingly stringent requirements which are imposed on engineering materials. Aluminum hydroxide and magnesium hydroxide are most often used flame retardants for this purpose. The latter is used more often in the processing of unplasticized PVC due to its higher decomposition temperature [19]. Magnesium hydroxide also contributes to a considerable reduction of the amount of generated smoke and lightening of its color during a fire, and makes the smoke less corrosive because it also scavenges the hydrochloric acid which is formed during combustion [20]. In addition to unplasticized PVC, plasticized blends with considerably reduced fire resistance due to the presence of a plasticizer are also used for many applications. In this case, it is especially desirable to use flame retardants [21].

Poly(vinyl chloride) is sensitive to heat and under processing conditions may undergo mechano-thermal destruction processes. These processes primarily involve progressive dehydrochlorination and may proceed following different mechanisms, which depend on the used thermal stabilizers [22]. Different types of stabilizers are used based on the applied PVC processing methods. These stabilizers are introduced either individually or, more often, in the form of systems characterized by a composition which causes a stabilizing synergistic effect [23,24]. Despite a relatively rich thermal stabilizer offer, the selection of an appropriate stabilizer that will meet the user-specified criteria for finished products is not easy. This is especially true for application areas, such as the packaging of food, toys or medical supplies.

Aside from standard stabilizers, the literature also describes an advantageous effect of other additives, including fillers used in PVC compounds, which can simultaneously enhance their dynamic thermal stability [13,25]. Folarin and Sadiku [22] reported a possibility of using hydrotalcite, also known as layered double hydroxide (LDH), as an additive which enhances the thermal stability of PVC and has a favorable effect on the reduction of smoke emission during PVC flaming. The stabilizing mechanism of LDH, found based on stability tests performed by the Congo red and TGA methods, was explained by weakening the activity of chlorine atoms and limiting the initiation of dehydrochlorination reaction [26,27]. The stabilizing effect of the layered double hydroxide (MgAlLDHs) was explained by the reaction of hydroxyl groups with hydrogen chloride, which limits the progress of PVC degradation [26].

The literature contains numerous examples of the use lignin and lignin-based materials as a filler of a polymer matrix, e.g., polyethylene, polypropylene, polystyrene, poly(ethylene terephtalate) or poly(ethylene oxide), in which lignin can act as a stabilizer against UV degradation or thermo-oxidation [28,29]. It is worth emphasizing that there is little information concerning the use of lignin as reinforcement in polymers such as PP, PE and PVC. Lignin is difficult to process since it cannot be heated to temperature greater than 190 °C, at which degradation begins to occur and mechanical properties are dramatically reduced [30,31]. The disadvantages of using lignin in composites include high water sorption, low elongation, difficulties related with flow through the injection nozzle and air bubbles. To overcome these limitations and enhance the potential of lignin fillers, different strategies have been realized, e.g., compatibilization of lignin–polymer composites [32,33], production of lignin graft copolymers [34] and use of lignin as a monomer for polymerization reactions [35,36].

Recently, much interest has been directed in the possibility of preparing functional fillers of silica combined with natural polymers, including lignin [37,38]. Silica, with its good mechanical and thermal resistance, can form interesting combinations with cheap and environmentally friendly lignin. New, functional hybrid materials may be used as polymer fillers [4,5,6,39,40]. The development of research focused on the search for new lignin-based hybrid fillers is justified. The following study directly corresponds with the above-mentioned field of research.

The results of our previous research regarding the use of silica/lignin fillers showed a favorable effect of lignin as one of the hybrid filler components on the thermal stability of PVC during processing by the method of kneading in a Brabender plastographometer [4]. It can therefore be presumed that introduction of a functional hybrid filler in the form of magnesium hydroxide with lignin to PVC dry blend may also contribute to an improvement of PVC thermal stability and, at the same time, it can be expected that such a filler may additionally reduce the combustibility of the PVC blend. In addition to the improvement of thermal properties and combustibility, the introduction of the hybrid filler with the proposed composition may contribute to a modification of the processing and mechanical properties of the composites. The assessment of the degree of those modifications is the main aim of the research undertaken within the present study.

## 2. Materials and Methods

### 2.1. Materials

Reagent-grade magnesium hydroxide (CAS number 1309-42-8) (Darmstadt, Germany) and kraft lignin (CAS number 8068-05-1)—average *M*_w_ ~10.000—both supplied also by Sigma Aldrich (Darmstadt, Germany), were used for the production of the hybrid filler. 

The Mg(OH)_2_/lignin hybrid material produced using the above-mentioned components was then used as the filler of composites on the matrix of unplasticized poly(vinyl chloride) (PVC) blend. In order to minimize the influence of processing additives, the stabilizer and the paraffin wax were the only additional components used in the PVC dry blend. The PVC dry blend was composed of: suspension PVC S-61 Neralit (Spolana Anwil Group, Neratovice, Czech Republic) 100 phr, organotin stabilizer Patstab 2310 (Patcham, Goor, The Netherlands) 4 phr, and 1 phr Naftolube FTP paraffin wax (Chemson, Arnoldstein, Austria).

The content of the hybrid filler (HM) in the PVC matrix amounted to 2.5 wt % (HM1), 5 wt % (HM2), 7.5 wt % (HM3) and 10 wt % (HM4), respectively. The obtained PVC composites contained the same percentage contents of Mg(OH)_2_, namely 2.5 wt % (M1), 5 wt % (M2), 7.5 wt % (M3) and 10 wt % (M4).

### 2.2. Preparation of Mg(OH)_2_/Lignin Hybrid Filler

In order to combine magnesium hydroxide and lignin, the process of mechanical grinding of pure precursors with their simultaneous mixing was employed using a Pulverisette 6 Classic Line planetary ball mill (Fritsch, Idar-Oberstein, Germany). The vessel with the materials subjected to homogenization (Mg(OH)_2_:lignin mass ratio equal to 1:1) was placed eccentrically on the rotary base of the planetary ball mill. The base rotation direction was opposite to the vessel rotation direction, with a speed ratio of 1:−2. The movement of agate balls within the vessel is the result of the action of the Coriolis force. The speed difference between the balls and the vessel results in the interaction of friction and impact forces, which generate high dynamic energy. The interaction of these two phenomena leads to a very high degree of size reduction of the ground/homogenized material. The mill operated with a change in rotation occurring every 5 min. To achieve the appropriate homogeneity of the final material, the grinding process was continued for 2 h. To prevent a possible overheating of the material due to continuous grinding, the mill automatically shut down every 30 min for 5 min, after which it resumed operation. Upon appropriate grinding, the hybrid material was separated through a 40 μm-mesh sieve.

### 2.3. Physicochemical and Dispersive–Morphological Characteristics of Inorganic–Organic Hybrid Filler and Used Components

#### 2.3.1. Scanning Electron Microscopy

The morphology and microstructure of the examined systems were assessed using an EVO40 scanning electron microscope manufactured by Zeiss (Oberkochen, Germany). Before testing, the samples were coated with Au for a time of 5 s using a Balzers PV205P coater (Oerlikon Balzers Coating SA, Brügg, Switzerland).

#### 2.3.2. Particle Size Distribution and Dispersive Parameters

The dispersion properties were determined by measuring the particle size in the range of 0.6–6000 nm, using a Zetasizer Nano ZS analyzer supplied by Malvern Instruments Ltd. (Worcester, UK). The operation of the analyzer is based on the Non-Invasive Back Scatter (NIBS) technique. The measurement of the size of particles suspended in a dispersing medium is possible owing to the fact that the particles are suspended in the liquid and are in constant motion due to the Brownian movements occurring as a result of their accidental collisions with the particles of the surrounding medium.

In addition, to determine the dispersive properties, a Mastersizer 2000 analyzer supplied by Malvern Instruments Ltd. (Worcester, UK) was used. The measurement relies on the technique of laser diffraction in the particle size range of 0.2–2000 μm. An important element of the apparatus is the Hydro 2000 G attachment, to which distilled water is poured to play the role of a dispersing medium for the measurement of the electric and optic backgrounds and also for minimizing the measurement errors caused by impurities. Before measurements the ultrasonic treatment was additionally applied.

#### 2.3.3. Thermogravimetric Analysis

The thermal stability analysis was made using a Jupiter STA 449F3 analyzer supplied by Netzsch GmbH, (Selb, Germany), which utilizes the thermogravimetry (TGA) method. The measurement involved heating a sample of an appropriate mass at the temperature range of 30–1000 °C with a step of 10 °C/min, in a nitrogen atmosphere. The analysis was performed by using the TGA-DTA attachment.

#### 2.3.4. Porous Structure Characteristics

The examination of the properties of the porous structure was made using an ASAP 2020 apparatus supplied by Micromeritics Instrument Co. (Norcross, GA, USA). The determination of the porous structure parameters was conducted using low-temperature nitrogen sorption. In the final examination phase, using suitable software, the most important parameters of the porous structure were determined, including the BET surface area (determined by the Brunauer–Emmett–Teller method) and the total volume and size distribution of pores (using the Barret–Joyner–Halenda (BJH) algorithm).

### 2.4. Preparation of Mg(OH)_2_/Lignin-PVC Composites

At the first stage of preparing composites in the Brabender mixer, compounds of the hybrid filler, previously dried for 3 h at the temperature of 105 °C, were prepared with a PVC dry blend. The homogeneous mixtures were processed in the chamber of a Brabender plastographometer (Plasti-Corder Pl 2200-3 type, Brabender GmbH & Co., Duisburg, Germany) at the temperature of 180 °C and a rotor speed of 30 rpm; the charge mass was 62 g. Kneading was conducted up to the point of establishing torque equilibrium, i.e., for about 10 min. The PVC mixtures with Mg(OH)_2_ as well as an unfilled PVC dry blend, used as the reference materials, were processed under identical conditions.

After cooling down, the processed material was ground and pressed using a hydraulic press which was constructed in the UTP Department of Technology of Polymer and Protective Coatings (the dimensions of the heating tables were 200 mm × 200 mm, the diameter of the piston was equal to 125 mm) at the temperature of 180 °C, under a pressure of 10 MPa for a duration of 5 min into 10 × 10 × 4 mm and 10 × 10 × 2 mm plates, which were used for the preparation of samples for mechanical testing and softening temperature determination by the Vicat method as well as for flammability test by the oxygen index method. The residue was ground and the obtained milling was used for thermal stability examination by the TGA and Congo red methods and for the determination of the mass flow rate.

### 2.5. Composite Characteristics

#### 2.5.1. Processing Properties

During kneading of the compounds, the torque of the rotors was recorded, as a function of time; simultaneously, the actual charge mass temperature was registered. Based on the obtained plastograms, the values characterizing the processing properties were determined, namely: the maximum value of the torque (*M*_X_), the value of the actual temperature of kneaded mixture (*T*_X_) associated with the maximum torque and the time of its attainment (*t*_X_), as well as the torque in the equilibrium state (*M*_E_) and the temperature of the mixture in the equilibrium state (*T*_E_).

Examinations of the mass flow rate (MFR) of the produced composites were also carried out using a D4004DE apparatus (Dynisco, Morgantown, VW, USA). The measurements were taken under the following conditions: cylinder temperature, 190 °C; piston load, 211.8 N; and length and diameter of the nozzle, 8.000 ± 0.025 mm and 2.095 ± 0.005 mm, respectively.

#### 2.5.2. Mechanical Properties

The mechanical properties were determined in accordance with ISO 527-1 using a Zwick Roell Z010 testing machine manufactured by Zwick GmbH & Co. KG, Ulm, Germany. The test was conducted using 1B-type 2 mm-thick specimens; 10 specimens from each material series were used for the determination.

#### 2.5.3. Thermal Properties

Thermogravimetric analysis and Congo red test were used to investigate the thermal stability of the samples. The TGA measurements of all samples were performed using a thermogravimetric analyzer Netzsch TG 209F3 (Selb, Germany), at a scanning rate of 10 °C/min under nitrogen atmosphere at the temperature range 20–900 °C.

Congo red thermal stability was determined at the temperature of 200 °C, according to ISO 182-1:1990.

The tests of Vicat softening temperature were performed in accordance with ISO 306:2014, using specimens 10 × 10 × 4 mm^3^. The measurements for one type of sample were carried out for 3 times, in accordance with the requirements of the standard.

The flammability test was performed by the oxygen index method at room temperature in accordance with Standard ISO 4589-2:2006. The determination of the lowest concentration of oxygen in its mixture with nitrogen, at which the burning of vertically positioned test specimens is maintained, was performed using 4 mm-thick specimens.

#### 2.5.4. Microscopic Observation

The structure of the PVC composites and the homogeneity of dispersion of the fillers was assessed by the optical microscopy method using a Nikon Eclipse 400 polarization microscope (Tokyo, Japan) at a magnification of 10 times. Approximately 0.1 mm-thick polymer films, obtained by the pressing method, were used for observations. Additionally, the microstructure was observed by scanning electron microscopy EVO40 (Zeiss, Oberkochen, Germany). The samples were broken in liquid nitrogen, and coated with gold for 5 s using a Balzers PV205P coater (Oerlikon Balzers Coating SA, Brügg, Switzerland) before placing in the chamber of the microscope.

## 3. Results

### 3.1. Physicochemical and Dispersive–Morphological Characterization of Mg(OH)_2_/Lignin Hybrid Filler

**Figure 1:**
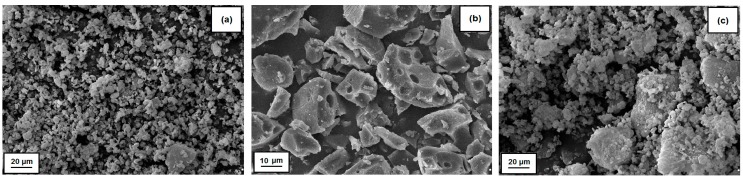
SEM images of: pure Mg(OH)_2_ (**a**); lignin (**b**); and Mg(OH)_2_/lignin hybrid filler (**c**).

Based on the observation made using SEM microscopy (Figure 1a), it can be found that magnesium hydroxide used for the preparation of hybrid system contains single primary particles, which show a tendency to form aggregates. Lignin, in turn, is characterized by a more uniform microstructure, which includes particles of sizes in the order of several dozen micrometers, with a pore-rich surface. In addition, this biopolymer exhibits a tendency to aggregate and agglomerate. The confirmation of the conclusions drawn from the analysis of a SEM image of lignin is provided by the dispersion data summarized in Table 1. These data show that the average lignin particle size, after the completed grinding process, is 19.8 μm, as indicated by parameter D(4.3) obtained from dispersion examinations carried out using a Mastersizer 2000 apparatus. Similarly, the presence of larger particles was observed based on the analysis of data obtained from the Zetasizer Nano ZS apparatus (see Table 1). The Mg(OH)_2_/lignin hybrid system, obtained based on the precursors used, contains particles in the size range of 342–615 and 955–5560 nm in its microstructure (the data from the Zetasizer Nano ZS apparatus). The data obtained with the Mastersizer 2000 analyzer and the SEM microphotograph (see Figure 1c) also confirm the presence of agglomerate forms in the hybrid structure. It can be observed that 50% by volume of Mg(OH)_2_/lignin hybrid system is occupied by particles with diameters smaller than 5.1 μm, while 90% of the sample volume is taken up by particles with diameters smaller than 9.7 μm. The average particle size in the hybrid system is 4.9 μm (see Table 1). In addition, magnesium hydroxide particles homogeneously covering lignin can be seen in Figure 1c.

**Figure 2:**
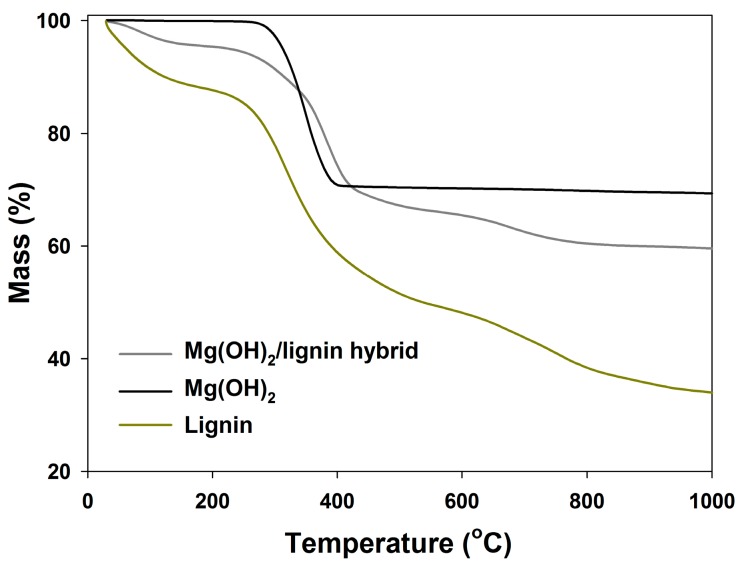
Thermal stability of magnesium hydroxide/lignin hybrid filler and used precursors (pure Mg(OH)_2_ and lignin).

Thermogravimetric analysis was employed for assessment of the thermal stability and, additionally, for the determination of the potential directions of application of the obtained hybrid material as a poly(vinyl chloride) filler. In Figure 2, the diagram of the relationship of the mass loss to temperature for the precursors used and the produced hybrid filler is shown. From the examination of the obtained curves, it can be inferred that magnesium hydroxide exhibits a mass loss of 30% in a temperature range of up to 1000 °C. The rapid decrease in Mg(OH)_2_ mass occurs at the temperature of 350 °C and is associated with the decomposition of magnesium hydroxide to magnesium oxide and with the elimination of the physically bonded water present in the compound under consideration, as confirmed in literature [41,42,43]. Lignin, in turn, shows a sample mass loss of 65% at a temperature range of up to 1000 °C. That considerable mass loss results from proceeding oxidation reactions, which result in the decomposition of the alkyl chains of the biopolymer. The first state corresponds to a small mass loss (at the temperature range from 30 to 200 °C) and is linked mainly to the localized elimination of water bonded on the lignin surface, which was also found in the papers [30,31]. The second stage of a considerable mass loss of approximately 40% at the temperature range from 200 to 600 °C is associated with the thermal decomposition of the compound, which involves the formation of new bonds as a result of occurring crosslinking reactions. Further heating of the sample above 600 °C, at the third stage, results in a sample mass loss of 15%, which is due to a partial elimination of the lignin fragments bonded with the aromatic carbon. In the sample remaining after heating, one can expect aromatic groups, which exhibit greater thermal stability compared to the alkyl substituents [5,39,44,45]. The performed thermal analysis of the prepared inorganic–organic hybrid filler allows concluding that the addition of magnesium hydroxide to lignin causes an improvement of thermal stability of the finished product. The examination of the thermogravimetric curve (see Figure 2) shows that the mass loss for the hybrid material amounted to 38% (in the range up to 1000 °C). The prepared hybrid system started to gradually decompose at the temperature of ~260 °C. The TGA results for the hybrid filler and both its components are presented in Table 2, which includes the temperature values corresponding to the mass loss of 5% and 50%, as shown in the study of Blanco et al. [46].

In order to obtain an in-depth assessment of the physicochemical properties of the obtained hybrid and the precursors used for its fabrication, the characterization of porous structure parameters was carried out (see Table 3). The hybrid filler used in the research was distinguished by a BET (Brunauer–Emmett–Teller) surface area of 73 m^2^/g and an overall pore volume and a pore size of 0.06 cm^3^/g and 4.2 nm, respectively. The precursors used, in turn, were characterized by a BET surface area of 108 m^2^/g (magnesium hydroxide) and 1 m^2^/g (lignin), which is consistent with the data previously determined and reported by other researchers [5,41].

### 3.2. Magnesium Hydroxide/Lignin-PVC Composite Characterization

#### Processing Properties

**Figure 3:**
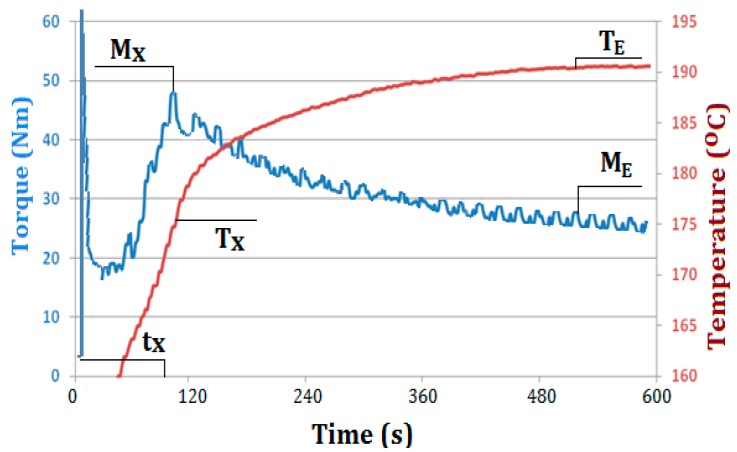
An exemplary plastogram of PVC/HM2 with characteristic values: *M*_X_, the maximum torque at the gelation point; *T*_X_, temperature at the gelation point; *t*_X_, time required to reach the *M*_X_; *M*_E_, torque at the end point; *T*_E_, temperature at the end point.

Figure 3 shows an exemplary plastogram of kneading of PVC mixture containing 5 wt % Mg(OH)_2_/lignin (PVC/HM2) with marked characteristic values, which were the subject of further analysis. The values of torque (M_X_ and M_E_), temperature (*T*_X_ and *T*_E_) and time (*t*_X_), determined from the plastograms, are summarized in Table 4.

The behavior of the dependency of the torque and the actual charge temperature on kneading time for all produced PVC composites are essentially similar and typical for unplasticized PVC blends (see Figure 3). The course of the plastograms, in which a distinct maximum of torque occurs, indicates correctly selected kneading conditions, i.e., temperature, the rotor speed and the charge mass, which determine the correct transformation of morphological structure of the PVC grains and the formation of a gelated material at the final kneading stage [47,48].

Compared to the kneading of unfilled PVC, the value of the maximum torque (M_X_), associated with PVC gelation, is higher and slightly increases along with the increase of the concentration of the hybrid filler in the kneaded blends (see Table 4). The addition of Mg(OH)_2_ to PVC caused a slight increase of the torque values compared to results obtained when the hybrid filler was used. Therefore, the presence of lignin in the hybrid filler slightly decreases the maximum torque of kneaded mixtures. A similar effect was observed during kneading of PVC mixes containing the silica/lignin hybrid filler [4,40], although, the reduction of the torque resulting from the presence of lignin was more effective due to the large torque value for the PVC/silica mixtures.

The time of attaining the maximum torque value is significantly shortened with increasing hybrid filler concentration. The shortest gelation time (shorter by approximately 55% compared to PVC) was found in case of mixture containing 10 wt % of the lignin hybrid filler. A lower reduction of the gelation time with increasing filler content of the matrix was observed during kneading of PVC mixtures with Mg(OH)_2_. The shortest gelation time (shorter by 25% compared to the unfilled PVC) was noted for mixtures containing 10 wt % of the filler.

Similar effects observed during kneading of PVC containing lignin modified with polyacrylate [49,50] as well as in our previous studies on PVC composites with the silica/lignin filler [4,40] might be caused by a change in densification of the processed PVC compounds, associated with the presence of the hybrid filler. The increase in the filler content contributes to an increase in the compaction of the kneaded PVC compound, an increase in shearing stresses and, as a consequence, an increase in torque and shortening in gelation time. Moreover, the presence of the filler may facilitate the heat transfer between PVC macromolecules and effectively accelerate their mutual penetration, which influences reduction in T_X_ temperature. The shortening of the gelation time and the decrease of the T_X_ temperature by 3–5 °C along with increasing hybrid filler content in the PVC matrix is advantageous from the economical point of view and the potential manufacturing of such composites in industrial practice. The analysis plastograms (see Table 4) also shows that the addition of the hybrid filler to kneaded PVC mixture does not practically cause any changes in the equilibrium-state torque, M_E_, and in the final actual temperature of the melted blends, similarly as in case of kneading compounds containing magnesium hydroxide. Therefore, no significant effect of the filler used in the amount of up to 10 wt % on the value of torque of kneaded PVC composites in melted state (M_E_) directly related to their viscosity is observed.

**Figure 4:**
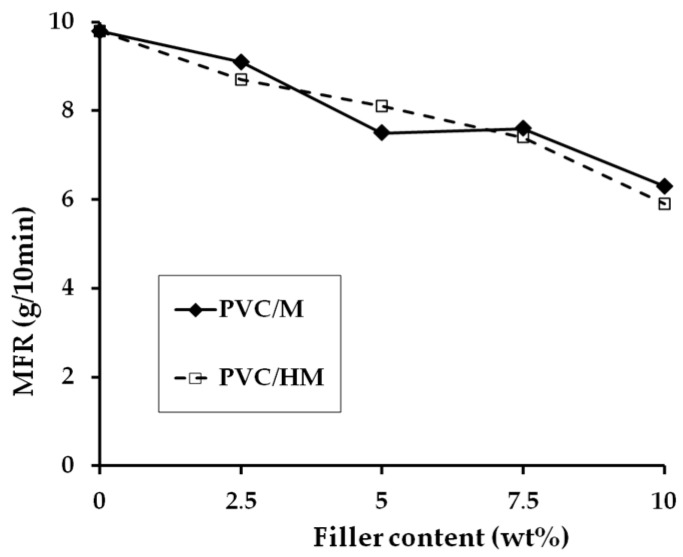
The value of MFR of PVC and PVC composites as a function of filler concentration.

The analysis of data in Figure 4 shows that the addition of a hybrid filler as well as its components, namely lignin and magnesium hydroxide, one at a time, to the PVC matrix, causes a slight reduction in the value of the composite mass flow rate, compared to unfilled PVC. Introduction of 2.5 wt % hybrid filler results in a decrease of MFR by 18%. Increasing the hybrid filler content in the PVC matrix to 10 wt % causes a further, steady decrease in MFR. The MFR values of PVC composites with Mg(OH)_2_ are approximately the same as the MFR values of the composites with hybrid fillers. A similar tendency of lowering the MFR value of the composites with PVC was found when lignin was used as a filler [4].

The reduction of the MFR value with the increase in the filler content of the PVC matrix is most likely caused by hindering the free movement of PVC macromolecules in the capillary of the plastometer due to the effect of the applied piston pressure. Such a possibility is confirmed by the relationship of the torque in the equilibrium state as a function of the increasing filler content (regardless of its type) shown in Table 4, although the observed changes of *M*_E_ values during kneading in the plastograph chamber are relatively negligible.

The analysis of test results of mechanical properties summarized in Table 5 indicates that the introduction of the used hybrid filler and, separately, magnesium hydroxide to PVC causes an increase in the modulus of elasticity at tension, the value of which increases along with increasing filler content in the matrix. In each case, the Young’s modulus of the composites is greater than the E_t_ value for unfilled PVC. During mechanical testing at static tension, it was found that specimens of all materials, i.e., PVC, PVC/M and the PVC/HM composites, indicated a distinct yield point. Regardless of the filler content, the value of the yield stress (*σ*_Y_) in each case corresponded to the tensile strength (*σ*_M_).

The tensile strength of the PVC/HM composites is lower than the σ_M_ of unmodified PVC and PVC/M composites, and its value decreases with increasing hybrid filler concentration. Furthermore, the modification of PVC with the hybrid magnesium hydroxide/lignin filler leads to a slight decrease of the mechanical strength of the composites, which was also observed in our previous studies, in which both silica/lignin hybrid fillers and a single component in the form of lignin were used [4,40]. This is a typical phenomenon for thermoplastic materials filled with both natural fibres and selected fillers with spherical particles and also applies to characteristics, such as impact resistance and relative elongation at break [51,52,53]. The hybrid composites obtained in this study are still characterized by beneficial strength which makes such materials suitable for application as engineering materials, in spite of the reduced σ_M_ value compared to PVC.

Regardless of their filler content, all PVC/HM and PVC/M composites showed a much lower elongation at break, compared to unfilled PVC, moreover the *ε*_B_ value of the PVC/HM composites is slightly lower in comparison to the composites modified only with magnesium hydroxide.

**Figure 5:**
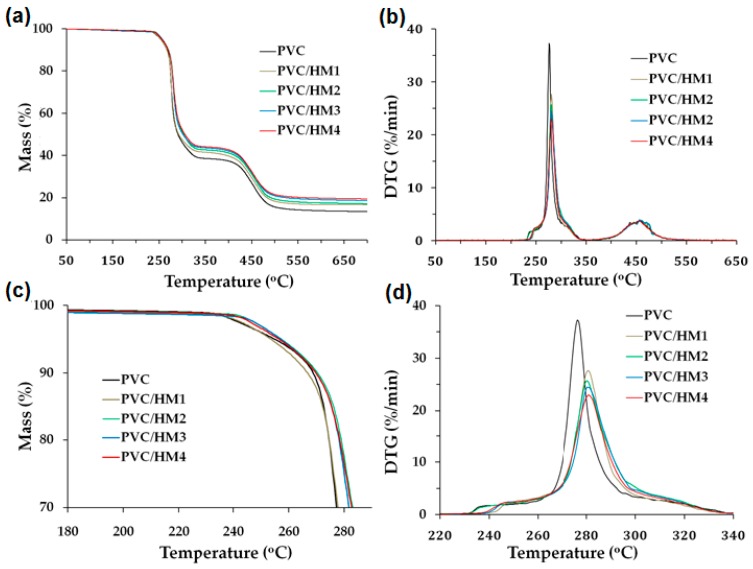
Thermograms for PVC and PVC/HM composites at various temperature range: (**a**) TGA curves (50–700 °C); (**b**) DTG curves (50–700 °C); (**c**) TGA curves (180–290 °C); and (**d**) DTG curves (220–340 °C).

**Figure 6:**
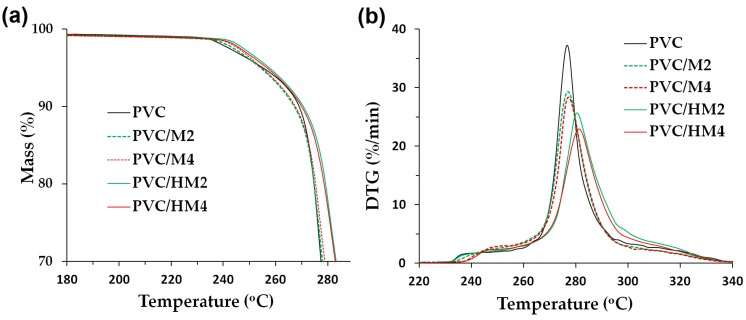
Thermograms for PVC as well as selected PVC/HM and PVC/M composites at various temperature range: (**a**) TGA curves (180–290 °C); and (**b**) DTG curves (220–340 °C).

Figure 5 presents TGA thermogram curves for the PVC/HM composites and the reference material, which were used to determine the values of the temperature *T*_5_ and *T*_50_ associated with the mass loss 5% and 50%, respectively, and the value of the temperature T_DTG_, at which the decomposition rate is maximal, similar to Blanco et al. [46]. Figure 6 presents a comparison of selected TGA and DTG thermogram curves for PVC/HM and PVC/M with 5 wt % and with 10 wt % of both types of fillers. Table 6 also summarizes other thermal properties of the obtained composites, such as thermal stability (determined with the Congo red test) as well as the values of the softening temperature and the oxygen index.

The behavior of the TGA curves for the PVC/HM composites (Figure 6a) and PVC/M composites is similar and typical for unfilled PVC. The decomposition of PVC proceeds in two stages. During the first stage, at the temperature range of 260–330 °C, the autocatalytic release of hydrogen chloride takes place, with the simultaneous formation of double bonds in the polymer chain. The released HCl additionally catalyzes and accelerates the reactions of destruction of PVC chains. The next stage of PVC decomposition, at the temperature range of 400–460 °C, involves the decomposition of the formed cross-linked polyene structure which results in residual chars [54].

The PVC composites with the hybrid filler (Figure 5c) and with magnesium hydroxide (Figure 6a), regardless of their content, are thermally stable within the processing temperature range, that is up to approximately 200 °C, as evidenced by the constant sample mass within this temperature range. In the case of unfilled PVC, the decomposition of the 5 wt % of the sample mass occurs at the temperature of approximately 255 °C. The addition of the Mg(OH)_2_/lignin filler slightly increases this temperature to a maximum value of 260 °C upon introducing 10 wt % of the hybrid to the matrix. In contrast, the value of temperature of the 5% decomposition for PVC/M is higher than for the unmodified PVC and the PVC/HM composites, which is associated with the higher thermal stability of Mg(OH)_2_ compared to lignin (see Figure 2). It should, however, be noted that the 5% mass loss of PVC/HM composites occurs only at the temperature range above 255 °C, i.e., at a much higher temperature than the processing temperature.

The hybrid filler also contributes to the increase of the temperature, at which 50% of the mass is decomposed (by 14 °C for the PVC/HM4 composite in comparison with unfilled PVC). Compared to PVC composites containing a single component of the hybrid, i.e., Mg(OH)_2_, PVC/HM composites are characterized by a higher value of temperature *T*_50_ (see Table 6). It can therefore be presumed that lignin contained in the employed hybrid filler has the effect of increasing the 50% sample mass loss temperature, which has also been found in our previous work [4].

In the DTG curve (Figure 5b), two separate endothermic peaks occur, the first of which represents the rate of the release process of HCl. The maximum of this wide peak indicates the value of the *T*_DTG_ temperature, at which the dehydrochlorination process takes place with the highest intensity. This temperature was approximately 276 °C for pure PVC, and with the increase of the Mg(OH)_2_/lignin filler content in the matrix, it shifts towards higher values, as shown in Figure 5d, reaching a value of 284 °C in the case of the PVC/HM4 composite. The second, smaller endothermic peak (see Figure 5b) is associated with further destruction of the material.

Compared to PVC composites modified with magnesium hydroxide, PVC/HM composites are characterized by a higher DTG peak temperature (Figure 6b) by approximately 3 °C for samples with a 5 wt % filler concentration and approximately 8 °C with a 10 wt % filler concentration.

Moreover, the height of the dehydrochlorination peak on the DTG curve, which indicates the intensity of HCl emission, decreases with the increase of both hybrid filler and Mg(OH)_2_ content in the PVC matrix. The maximum PVC decomposition rate is approximately 37%/min, introduction of 10 wt % of the magnesium hydroxide reduces this value to 28%/min. Introduction of 10 wt % of the Mg(OH)_2_/lignin hybrid filler reduces this value to 23%/min. Thus, the Mg(OH)_2_/lignin hybrid material proposed in this work has an advantageous contribution to reducing the intensity of HCl emission during the decomposition of PVC used as the matrix of PVC/HM composites.

Similar absorption properties of HCl and thus stabilization properties were found in the case of using micrometer and nanometer CaCO_3_ as a filler in PVC composites produced by the solution casting method [25]. The TGA analysis shows that both the decomposition starting temperature and the temperature of maximum degradation rate are higher along with the increase of filler concentration in the matrix. The authors have concluded that the CaCO_3_ particles can bind the HCl gas, whereby the self-accelerating effect is avoided when CaCO_3_ is used as a filler. Scavenging of HCl does not lead to a complete stop of the decomposition process, though it can significantly reduce the rate of degradation. The stabilizing effect of magnesium hydroxide due to the absorption of hydrogen chloride released during PVC degradation, which has a direct effect of extending the duration of thermal stability, as measured by the Congo red method, is also described [55]. The effective activity of hydrotalcite (LDH), which includes, inter alia, magnesium hydroxide and aluminum hydroxide, as the modifier of thermal stability and the reducer of smoke emission during PVC flaming, is explained by authors of studies [26,56,57,58,59] as the absorption and, consequently, reaction between LDH and gaseous HCl evaporated from PVC, which leads to the formation of metal chlorides.

A potential application of lignocellulose materials as the absorbers of hydrogen chloride released during destruction of polymer medical wastes including PVC was proposed in study [60]. The mechanism of the reaction of HCl absorbed by lignin with the methoxy groups in phenolic rings, which leads to the formation of chloromethane, was explained by Czégény et al. [61]. This reaction consumes evaporated HCl which is released during the PVC decomposition.

The filler used in this study contains both of the components described in the literature cited above, i.e., Mg(OH)_2_ and lignin, hence it can be presumed that the mechanism of the stabilizing effect of this filler on PVC is complex and may result from the simultaneous interactions of both hybrid components with polymer macromolecules.

The favorable effect of the used filler on the static thermal stability is also confirmed by the results of the Congo red method. The analysis of the data presented in Table 6 shows that introduction of the Mg(OH)_2_/lignin filler to the matrix in the amount of 5 wt % and more significantly extends the time after which the change in the indicator’s color is observed, which confirms the destruction of PVC. PVC composites containing 7.5 and 10 wt % are characterized by higher temperature stability compared to unfilled PVC by 29 and 41 min, respectively. The time of the thermal stability of PVC/M composites increases with increasing Mg(OH)_2_ content of the matrix, though the stability improvement is not as considerable as in the case of the hybrid filler. It can therefore be inferred that the significantly increased thermal stability of PVC/HM is associated with the presence of the lignin component, as has also been found in work [4].

It can be presumed that the presence of lignin in the hybrid filler is an important factor preventing the chain reactions of PVC dehydrochlorination, probably due to attaching HCl molecules initiating the chain reactions of decomposition to metoxy groups in phenolic rings, which leads to the formation of chloromethane [61]. In addition, the participation of the Mg(OH)_2_ component reinforces this effect, the HCl released at the initial stage of destruction may be a substrate for the reaction of synthesis from Mg(OH)_2_, resulting in MgCl_2_ [19,20,62].

The PVC/HM composites are distinguished by a higher softening temperature compared to unfilled PVC and PVC/M composites (see Table 6); introduction of 2.5 wt % of the hybrid filler to the matrix causes the softening temperature to increase by 5 and 6 °C, respectively. Further increase in VST with growing hybrid filler concentration is less notable. The VST temperature of the composites modified with magnesium hydroxide is similar to that of the unmodified PVC regardless of the concentration of the filler.

A similar effect of improving the functional properties of PVC matrix-based composite materials in terms of widening their temperature applicability range is described for the use of the wood flour filler [44] and the SiO_2_/lignin hybrid filler [4].

Within the present study, a preliminary investigation was also carried out to determine the usefulness of the Mg(OH)_2_/lignin filler as an agent enhancing the fire resistance, as determined by the LOI method. The value of the LOI index of unfilled PVC sample is 45.1%, which allows it to be ranked among self-extinguishing materials. It has been found that the PVC/HM composites are characterized by LOI values ranging from 45.8% to 49.6%, which may suggest a potential for using the Mg(OH)_2_/lignin hybrid as an agent which additionally increases the fire resistance of PVC.

As expected, the LOI value of PVC composites containing 10 wt % of Mg(OH)_2_ is higher by approximately 20% compared to pristine PVC and by approximately 11% compared to a PVC sample containing the identical hybrid filler fraction.

Within this study, the examination of the LOI of PVC composites with a lignin addition was also carried out. The LOI values of composites are similar and range from 43.6% to 44.0% regardless of the lignin content up to 10 wt %. Thus, the increase in the oxygen index of PVC-based composites is associated with the presence of an inorganic component in the hybrid filler.

**Figure 7:**
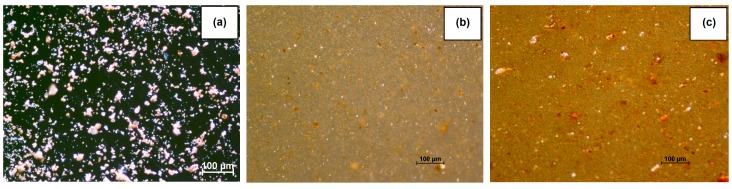
The optical microscopy images of hybrid filler (**a**); and PVC composites with hybrid filler: 2.5 wt % (**b**); and 10 wt % (**c**).

Figure 7 shows microscopic images of filler particles and the structure of the PVC/HM1 and PVC/HM4 composites taken in reflected light. In case of the PVC/HM1 composite (Figure 7b), uniformly dispersed 5–12 µm hybrid filler particles within the PVC matrix were observed. While the content of the filler in composite was at 10 wt %, its homogeneous distribution (Figure 7c) was also observed, however, isolated 20–50 µm particle aggregates are visible in the picture.

**Figure 8:**
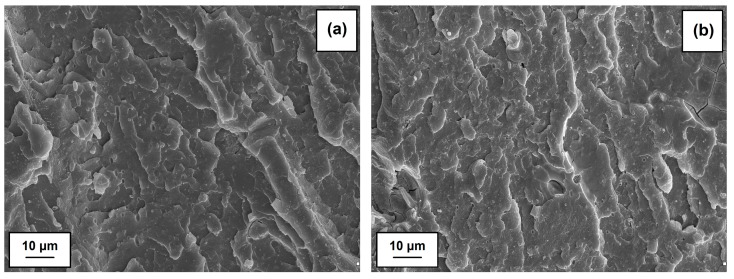
SEM images of PVC composites with hybrid filler: 2.5 wt % (**a**); and 10 wt % (**b**).

SEM images (see Figure 8) confirm the homogeneous dispersion of the hybrid filler in the PVC matrix. Moreover, the surface of fractures indicates a layer structure characteristic of gelated PVC, which verifies that the conditions of composites processing were properly chosen. Based on SEM observations, it may be stated that the adhesion between the PVC matrix and hybrid filler is sufficient; the phase separation is not observed. Similar morphology was also noticed for polypropylene composites with the mixture of cellulose, hemp, fax and lignin [63].

## 4. Conclusions

Within the study, a novel, functional Mg(OH)_2_/lignin hybrid system has been produced by a mechanical method using a planetary ball mill and described. The obtained material is distinguished by an average particle size of 4.9 μm and a BET surface area of 73 m^2^/g. In addition, the potential for using the inorganic–organic hybrid as a possible PVC filler has been confirmed by thermal stability analysis, which allowed determining that the total mass loss at the temperature range up to 1000 °C amounts to 38%.

The addition of the Mg(OH)_2_/lignin hybrid filler has a favorable effect on the processing properties of composites on the unplasticized PVC blend by shortening of the gelation time. In contrast, no significant increase in the mechanical and thermal loads of the processed compounds is observed. The same applies to the effect of the employed filler on the resistance of kneading the composites in a melted state, associated with their viscosity. In addition, introduction of the HM filler to the PVC causes a significant improvement of its thermal stability. The PVC composites with the Mg(OH)_2_/lignin filler are also characterized by good mechanical properties. The increase of the oxygen index of PVC composites due to the introduction of hybrid fillers with the proposed composition, which was observed in the study, may contribute to an extended range of their application under conditions that require special fire resistance.

In summary, the obtained results indicate the possibility of using the magnesium hydroxide/lignin filler as an additional component of PVC dry blends to improve the essential properties of composites produced from them. However, further studies are purposeful to determine the smoke generation of the PVC/HM composites and their other parameters, such as the quantity of heat released during combustion.

## Figures and Tables

**Table 1:** Dispersive properties of inorganic–organic hybrid filler as well as pure magnesium hydroxide and kraft lignin.

Sample Name	Dispersive Properties				
	Particle Size Distribution from Zetasizer Nano ZS (nm)	Particle Diameter from Mastersizer 2000 (μm)			
		d(0.1) *	d(0.5) **	d(0.9) ***	D(4.3) ****
Magnesium hydroxide	91–513	1.0	2.6	4.8	2.4
Kraft lignin	1110–5560	6.9	20.3	38.9	19.8
Mg(OH)_2_/lignin hybrid material	342–615; 955–5560	2.3	5.1	9.7	4.9

* d(0.1), 10% of the volume distribution is below this diameter value; ** d(0.5), 50% of the volume distribution is below this diameter value; *** d(0.9), 90% of the volume distribution is below this diameter value; **** D[4.3], average particle size in examined system.

**Table 2:** Thermal properties of the tested materials.

Sample Name	TGA Results	
	*T*_5_ * (°C)	*T*_50_ ** (°C)
Mg(OH)_2_/lignin hybrid	156	378
Magnesium hydroxide	287	343
Lignin	46	344

* *T*_5_, temperature at 5% mass loss; ** *T*_50_, temperature at 50% mass loss.

**Table 3:** Parameters of porous structure of Mg(OH)_2_/lignin hybrid filler and pure precursors.

Sample Name	Parameters of Porous Structure		
	BET Surface Area (m^2^/g)	Total Volume of Pores (cm^3^/g)	Mean Size of Pores (nm)
Magnesium hydroxide	108	0.10	2.6
Kraft lignin	1	0.01	11.4
Mg(OH)_2_/lignin hybrid material	73	0.06	4.2

**Table 4:** The fusion properties of the PVC composites.

Sample Name	*M*_X_ (Nm)	*T*_X_ (°C)	*t*_X_ (s)	*M*_E_ (Nm)	*T*_E_ (°C)
PVC	45	178	160	26	190
PVC/M1	45	175	135	25	191
PVC/M2	51	178	130	26	191
PVC/M3	51	178	130	27	191
PVC/M4	53	177	120	26	192
PVC/HM1	46	175	105	27	191
PVC/HM2	48	174	95	27	191
PVC/HM3	51	174	75	28	191
PVC/HM4	50	176	70	26	191

**Table 5:** Mechanical properties of the tested materials.

Sample Name	*E*_t_ (MPa)	*σ*_Y_ (MPa)	*σ*_M_ (MPa)	*ε*_B_ (%)
PVC	1590 ± 18.6	53.7 ± 0.9	53.7 ± 0.9	41.3 ± 11.7
PVC/M1	1620 ± 27.8	48.3 ± 2.5	48.3 ± 2.5	19.9 ± 11.9
PVC/M2	1770 ± 64.4	51.5 ± 1.5	51.5 ± 1.5	7.5 ± 2.9
PVC/M3	1780 ± 69.8	53.3 ±.2.1	53.3 ±.2.1	10.2 ± 0.9
PVC/M4	1810 ± 73.1	52.1 ±.2.2	52.1 ±.2.2	5.2 ± 0.8
PVC/HM1	1760 ± 38.2	50.4 ± 2.7	50.4 ± 2.7	5.3 ± 1.2
PVC/HM2	1750 ± 87.1	46.5 ± 3.2	46.5 ± 3.2	4.5 ± 0.9
PVC/HM3	1740 ± 75.4	43.0 ±.2.1	43.0 ± 2.1	3.6 ± 0.9
PVC/HM4	1780 ± 67.7	42.5 ±.3.8	42.5 ± 3.8	4.7 ± 0.8

**Table 6:** Thermal properties of the tested materials.

Sample Name	TGA Results			Thermal Stability (min)	VST (°C)	LOI (%)
	*T*_5_ * (°C)	*T*_50_ ** (°C)	*T*_DTG_ (°C)			
PVC	255.5	292.0	276.2	22	81.4	45.1
PVC/M1	274.3	293.0	274.3	21	80.1	47.8
PVC/M2	277.0	291.1	277.0	30	82.4	52.2
PVC/M3	279.6	301.8	278.6	36	80.7	53.0
PVC/M4	277.2	300.4	276.6	39	79.3	55.2
PVC/HM1	255.1	297.4	280.9	24	86.2	45.8
PVC/HM2	256.3	299.4	280.8	35	88.9	47.2
PVC/HM3	258.1	303.8	281.5	51	89.1	48.4
PVC/HM4	260.1	306.2	284.4	63	90.0	49.6

* *T*_5_, temperature at 5% mass loss; ** *T*_50_, temperature at 50% mass loss.
